# Rapid, high efficiency virus-mediated mutant complementation and gene silencing in *Antirrhinum*

**DOI:** 10.1186/s13007-020-00683-5

**Published:** 2020-10-27

**Authors:** Ying Tan, Alfredas Bukys, Attila Molnár, Andrew Hudson

**Affiliations:** 1 Institute of Molecular Plant Sciences, University of Edinburgh, Max Born Crescent, Edinburgh, EH9 3BF UK; 2 College of Life Sciences, Hunan Normal University, 136 Lushan Road, Changsha, 410006 China

**Keywords:** *Antirrhinum*, *Misopates*, VIGS, Tobacco rattle virus, TRV, Protein expression

## Abstract

**Background:**

*Antirrhinum* (snapdragon) species are models for genetic and evolutionary research but recalcitrant to genetic transformation, limiting use of transgenic methods for functional genomics. Transient gene expression from viral vectors and virus-induced gene silencing (VIGS) offer transformation-free alternatives. Here we investigate the utility of Tobacco rattle virus (TRV) for homologous gene expression in *Antirrhinum* and VIGS in *Antirrhinum* and its relative *Misopates.*

**Results:**

*A. majus* proved highly susceptible to systemic TRV infection. TRV carrying part of the *Phytoene Desaturase* (*PDS*) gene triggered efficient *PDS* silencing, visible as tissue bleaching, providing a reporter for the extent and location of VIGS. VIGS was initiated most frequently in young seedlings, persisted into inflorescences and flowers and was not significantly affected by the orientation of the homologous sequence within the TRV genome. Its utility was further demonstrated by reducing expression of two developmental regulators that act either in the protoderm of young leaf primordia or in developing flowers. The effects of co-silencing *PDS* and the trichome-suppressing *Hairy* (*H*) gene from the same TRV genome showed that tissue bleaching provides a useful marker for VIGS of a second target gene acting in a different cell layer. The ability of TRV-encoded H protein to complement the *h* mutant phenotype was also tested. TRV carrying the native *H* coding sequence with *PDS* to report infection failed to complement *h* mutations and triggered VIGS of *H* in wild-type plants. However, a sequence with 43% synonymous substitutions encoding H protein, was able to complement the *h* mutant phenotype when expressed without a *PDS* VIGS reporter.

**Conclusions:**

We demonstrate an effective method for VIGS in the model genus *Antirrhinum* and its relative *Misopates* that works in vegetative and reproductive tissues. We also show that TRV can be used for complementation of a loss-of-function mutation in *Antirrhinum.* These methods make rapid tests of gene function possible in these species, which are difficult to transform genetically, and opens up the possibility of using additional cell biological and biochemical techniques that depend on transgene expression.

## Background

*Antirrhinum* species (snapdragons, in the family Plantaginaceae) have provided models to study plant genetics, development and evolution for over a century [1, 2]. Their use is supported by infrastructure that includes genome sequences [3], transcriptomes [4], genetic maps [5, 6] and computational frameworks for growth analysis [7–9]. However, *Antirrhinum* has proved recalcitrant to genetic transformation. In other model plant species, efficient transformation methods allow gene functions to be tested in many different ways, for example by reducing gene function with RNAi or genome editing, mis-expression or complementation. Genetic transformation also underpins many cell biological and biochemical methods that allow gene and protein functions to be investigated further. Though transgenic *A. majus* plants have been produced by infecting hypocotyl explants with *Agrobacterium tumefaciens* and regenerating plants from transgenic cells, or by regeneration from root cultures transformed by *A. rhizogenes* [10–12], these methods involve tissue culture and regeneration and are therefore slow, as well as very inefficient [13, 14]. Consequently they have not been adopted widely.

To test the function of genes by reducing their activity, virus-induced gene silencing (VIGS) provides a potentially quicker alternative to methods that require genetic transformation. It involves triggering the plant’s defence mechanisms with a viral RNA genome carrying a sequence homologous to the target gene [15–17], resulting in RNAi-mediated destruction of both virus and target gene RNA. It can also result in RNA-directed DNA methylation, which can maintain the target gene in a silenced state through cell division, potentially giving rise to clones of cells with reduced expression [18, 19]. However, unlike stable null mutations, including those produced by genome editing, VIGS rarely results in complete silencing and its effectiveness can vary even between parts of the same plant, giving mosaics of cells with different expression levels [15, 18, 19]. Without a marker for VIGS, relating a phenotypic change to a reduction in gene expression can therefore be difficult. The use of VIGS is also potentially limited by the ability of the virus to infect cells in which the target gene is expressed—for example, most viruses are excluded from apical meristems, where many developmental regulator genes act [17].

Two examples of VIGS have been reported in *A. majus*. One used Cucumber mosaic virus (family Bromoviridae) to examine the role of *AmANT* [20], the *A. majus* orthologue of *AINTEGUMENTA*, which promotes cell division in the lateral organs of *Arabidopsis* [21–23]. An *AmANT* sequence was included in the tripartite RNA genome of CMV, which had been transcribed in vitro and used to infect *Nicotiana benthamiana*. Following inoculation of *A. majus* with sap from *N. benthamiana*, *AmANT* RNA was decreased significantly in flowers and leaf growth was reduced. Efficiency, in terms of the proportion of inoculated plants that experienced VIGS, was not reported. The second example used Tobacco rattle virus (TRV; a tobravirus in the family Virgaviridae) to reduce expression of the *AmSPB1* gene. The RNA genome of TRV was expressed directly in *A. majus* from T-DNAs delivered to seedlings by infiltration with a suspension of *A. tumefaciens* (Agro-infiltration). Only about one half of the treated seedlings survived, and virus could be detected in only around 2% of survivors [13]. However, *AmSPB1* RNA was reduced significantly, revealing that TRV is also capable of causing VIGS in *Antirrhinum*, albeit with low efficiency when delivered directly by Agro-infiltration.

RNA viruses have also been adapted to express heterologous proteins in plants [24]. Though used less commonly for this than some other RNA viruses, TRV has the advantages of a broad host-range [25, 26], causing only mild symptoms in many hosts and being able to infect apical meristems in some species [26, 27]. It has a bipartite, positive-strand RNA genome in which the smaller RNA, TRV2, encodes viral coat protein (CP) and the non-structural proteins *2b and 2c*, which are expressed from shorter, sub-genomic RNAs [17, 28, 29]. Both *2b* and *2c* genes appear dispensable for mechanical transmission and systemic infection [28, 29] and can be replaced with heterologous sequence, reducing the pressure that increased genome size might otherwise place on a recombinant virus [24, 30–32]. The larger genome, TRV1, which encodes the viral replicase, movement protein and a silencing suppressor, has usually been left intact [26]. While TRV has been used as both a VIGS vector and to express heterologous proteins, either as CP fusions or as unfused proteins [24], there are relatively few reports of viral vectors being used to express plant proteins, and most of these have involved sequences from a different plant species to the host (e.g., [33, 34]). However, complementation of a mutant phenotype in tomato fruits (ripening inhibited, rin) has been achieved by viral expression of a wild-type Rin protein from a *Potato virus X* vector (Alphaflexiviridae) [35, 36].

Here we test the ability of TRV-mediated VIGS to silence three *Antirrhinum* genes needed in different locations and at different stages of development. The first, *Phytoene Desaturase* (*PDS*), is necessary for synthesis of carotenoid antioxidants in photosynthetic tissues [37]. Because reduced *PDS* activity results in visible photo-oxidative bleaching of green tissues, it is often used as a reporter for VIGS [17]. The *Divaricata* (*Div*) gene, in contrast, encodes a transcription factor that is expressed in the developing flower, where it has a dose-dependent effect on dorsiventral asymmetry [38, 39]. The most obvious effects of reduced Div activity are loss of the characteristic shape of the ventral-most petal and of trichomes (hairs) found only inside the ventral corolla tube [40]. The third target, *Hairy* (*H*) encodes a glutaredoxin that is expressed only in the epidermis of leaf primordia and stems above metamer 4 (node 3 and the internode above it), where it is needed to suppress development of epidermal trichomes [41]. This function is conserved in other *Antirrhinum* species with a limited distribution of trichomes and in *Misopates orontium*, a fellow member of the tribe *Antirrhineae* that last shared an ancestor with *Antirrhinum* around 12 million years ago [42, 43]. H activity has been lost from some *Antirrhinum* species that produce trichomes from all leaf blades and stems [41]. Because such *h* mutants are already available and the *H* coding sequence is relatively short (324 bp) and lacks introns [41], we also used *H* to test whether protein expression from TRV could be used in complementing a mutant phenotype in *Antirrhinum*.

## Results

**Fig. 1:**
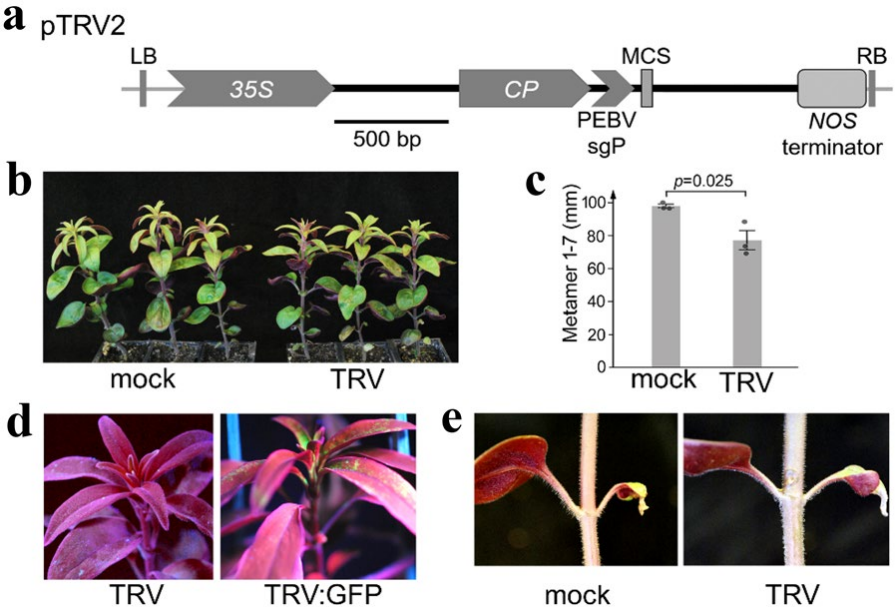
TRV infection in *A. majus*. **a** The T-DNA region of pTRV2sgP, delimited by left and right borders (LB, RB), which expresses the TRV2 RNA (black line) from a double *35S* promoter. A *GFP* ORF was inserted into the multiple cloning site (MCS) 3′ to a sub-genomic promoter (sgP) from Pea early browning virus (PEBV). TRV2sgP encodes coat protein (CP), but lacks *2b* and *2c* genes, which encode non-structural proteins. TRV1, which encodes the viral replicase, movement protein and 16 K silencing repressor, is not shown. **b** Representative plants that were either inoculated with virus in which the TRV2 genome was transcribed from empty pTRV2sgP (TRV) or mock-inoculated (mock). **c** Inoculation with TRV reduced plant growth (shown here for metamer 1–7 stem length, *p* = 0.025 from a Student’s *t*-test). **d** Plants inoculated with virus produced from pTRV2 that carried either a *GFP* ORF (TRV:GFP) or that did not (TRV). The two images were taken under UV illumination to detect GFP fluorescence. The inoculated leaves are below the ones shown in these two images. **e** Metamer 2 leaves 12 days after inoculation. The right-hand leaf in each image was rub-inoculated with infectious sap (TRV) or buffer only (mock)

Given the high mortality and low efficiency reported to result from direct Agro-infiltration of *A. majus* [13], we first produced infectious virus in *N. benthamiana* by infiltration with a mixture of *A. tumefaciens* strains carrying pTRV1, which expresses the TRV1 RNA, and either empty or recombinant pTRV2sgP, to express TRV2 RNA (Fig. 1a). After systemic infection of *N. benthamiana* had occurred, infectious sap was extracted and used to rub-inoculate *Antirrhinum* or *Misopates* plants at different stages of development.

### *A. majus* is susceptible to infection with recombinant TRV

Inoculating *A. majus* seedlings with TRV1 and empty TRV2sgP, led to slightly reduced growth and distortion of leaves compared to mock-inoculated control plants (Fig. 1b,c), suggesting that TRV was able to infect *A. majus*. Infection and systemic spread of the virus was confirmed with TRV2 carrying the coding sequence of GFP downstream of the heterologous sub-genomic promoter (sgP); GFP expression was seen within 6 days in 21 out of 22 inoculated plants (95%), even in leaves initiated several weeks after infection (Fig. 1d). Inoculated leaves and cotyledons often withered or died, though this was likely the result of mechanical damage because it also happened after mock inoculation (Fig. 1e). We therefore inoculated only one leaf and one cotyledon from each plant, to preserve photosynthetic source tissues that might aid spread of the virus to younger, sink tissues.

### Plants infected as seedlings can show persistent VIGS of *AmPDS*

**Fig. 2:**
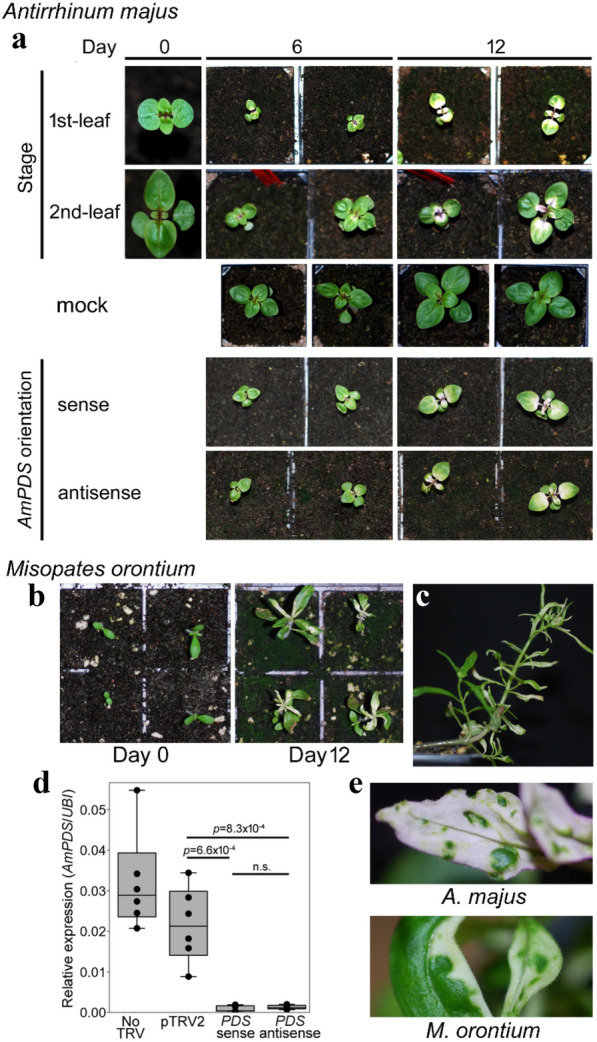
VIGS of PDS in *Antirrhinum* and *Misopates*. **a** *A. majus* plants were inoculated with TRV carrying an *AmPDS* fragment in antisense orientation at either the first-leaf stage or second-leaf stage (shown as Day 0). Plants at the first-leaf stage were also inoculated in a second experiment with TRV carrying the same *AmPDS* fragment in either sense or antisense orientation. Progression of VIGS (tissue bleaching) is shown in the same plants at 6 days and 12 days post inoculation with plants mock-inoculated at the 2-leaf stage shown below them for comparison. **b** Inoculation of *M. orontium* seedlings with TRV carrying the *AmPDS* antisense fragment caused bleaching within 12 days. **c** In around half of the *M. orontium* plants, VIGS of *PDS* persisted into the inflorescence (the shoot to the right). **d** TRV carrying *AmPDS* sequence reduced *A. majus* host *AmPDS* RNA abundance significantly, and sense and antisense orientation of *AmPDS* were equally effective. Values are the ratios of *AmPDS* to *Ubiquitin1* (*UBI*) RNA for three biological replicates with two technical replicates of each. Median and quartile values are indicated. Differences between means were detected with ANOVA and Tukey Post-hoc tests. **e** VIGS of *PDS* in both *A. majus* and *M. orontium* reduced growth of tissue relative to neigbouring tissue that had remained green

To provide a visual marker for VIGS, we identified the single-copy *AmPDS* gene in the *A. majus* reference genome [3] and amplified a cDNA containing the last 200 bp of coding sequence and 160 bp of the following 3′-UTR. The longest stretch of nucleotides that this sequence shared with the transcript of another gene was 18 bp, suggesting that the potential for off-target VIGS of other genes was minimal [44]. The cDNA fragment was cloned into pTRV2sgP, in either sense or antisense orientation relative to the TRV2 genome. TRV carrying *AmPDS* in antisense orientation was tested first. It was used to inoculate *A. majus* plants at three developmental stages: before the second pair of true leaves was clearly visible (first-leaf stage, ~ 21 days after germination; Fig. 2a), 1 week later (second-leaf stage), or 4 weeks later (fifth-leaf stage). Photo-bleaching, characteristic of reduced PDS activity, became visible by 6 days post inoculation (dpi) for plants infected at the two earlier stages. It was seen in leaves that had not been inoculated, consistent with systemic spread of TRV or VIGS, and became more obvious by 12 dpi (Fig. 2a). All 14 plants infected at the first-leaf stage showed VIGS, compared to 10 out of 14 plants inoculated at the second-leaf stage, suggesting that inoculation at the earlier stage may be marginally more effective at inducing VIGS (one-tailed Fisher’s Exact Test *p* = 0.049). No evidence of VIGS was found in 24 plants inoculated at the fifth-leaf stage.

### VIGS in *Misopates orontium*

*Misopates* is a likely sister genus to *Antirrhinum*, within the tribe *Antirrhineae*, that has been used for comparative studies [45, 46] but lacks a system for reverse genetics. We therefore tested whether the VIGS method for *A. majus* was directly transferable to *M. orontium*. Eighty *M. orontium* seedlings were inoculated at the first-leaf stage with the TRV carrying antisense *AmPDS*. At 12 dpi, bleaching could be seen in 75 out of the 79 plant that survived inoculation (Fig. 2b). This indicated that VIGS with TRV was also effective in this species and that the similarity between the viral *AmPDS* sequence and the *M. orontium PDS* target was sufficient to cause VIGS. Bleaching was lost from newly formed shoot tissue in some plants, but extended into the inflorescences of 37 plants (46% of those inoculated, Fig. 2c).

### The *AmPDS* sequence effectively triggers VIGS in both antisense and sense orientations

The antisense orientation of an homologous sequence within the viral genome has been reported to be more effective at causing VIGS than the sense orientation [47, 48]. To test this for TRV in *A. majus*, we used the same *AmPDS* sequence in opposite orientations and inoculated *A. majus* plants at the first leaf stage. For each treatment, bleaching was visible by 6 dpi in 48 plants out of the 50 inoculated (Table 1, Fig. 2a). VIGS continued in growing shoots to at least metamer 6 (node 5 and the internode above it) in about half of the plants. As in *M. orontium*, it was often lost from tissues that formed later. However, around one in five plants still showed VIGS in the inflorescence and flowers (Table 1). *AmPDS* RNA abundance was reduced significantly in the *AmPDS*-silenced plants and no difference was detected between the effects of the sense and antisense orientations of *AmPDS* (Fig. 2d). In both *A. majus* and *M. orontium*, bleached tissue grew less than adjacent unbleached tissue (Fig. 2e) and plant height was often reduced.

**Table 1:** Efficiency and persistence of VIGS in *A. majus*

Developmental stage	Orientation of insert		*P* value†
	Sense	Antisense	
Metamer 3	48,96%*	48,96%	1.00
Metamer 6	27,54%	26,52%	0.89
Inflorescence	12,24%	11,22%	0.83

Fifty *A. majus* plants were inoculated at the first leaf stage with TRV carrying *AmPDS* in either sense or antisense orientation. *The number and percentage of plants showing bleaching in metamer 3 or metamer 6 leaves or in the inflorescence (> metamer 10). ^†^Fisher’s Exact Test probabilities that the effects of antisense and sense orientations are not different

These results suggest that the orientation of the targeted gene sequence within TRV was not a major factor in the induction or persistence of VIGS. They also suggest that VIGS declines with age in both *A. majus* and *M. orontium* plants. This could be because the virus is less likely or less able to infect organs formed later in development, consistent with the inability of inoculation at a later (fifth-leaf) stage to cause VIGS, or because antiviral RNAi increases over time. Alternatively, heritable silencing of *PDS* could be induced at an early stage and lost progressively as plants grow. Regardless of the reason, persistence of VIGS in around one quarter of plants inoculated at the seedling stage made this method potentially useful to study the function of genes acting at any stage of *Antirrhinum* or *Misopates* development.

### VIGS of genes regulating floral or epidermal development

**Fig. 3:**
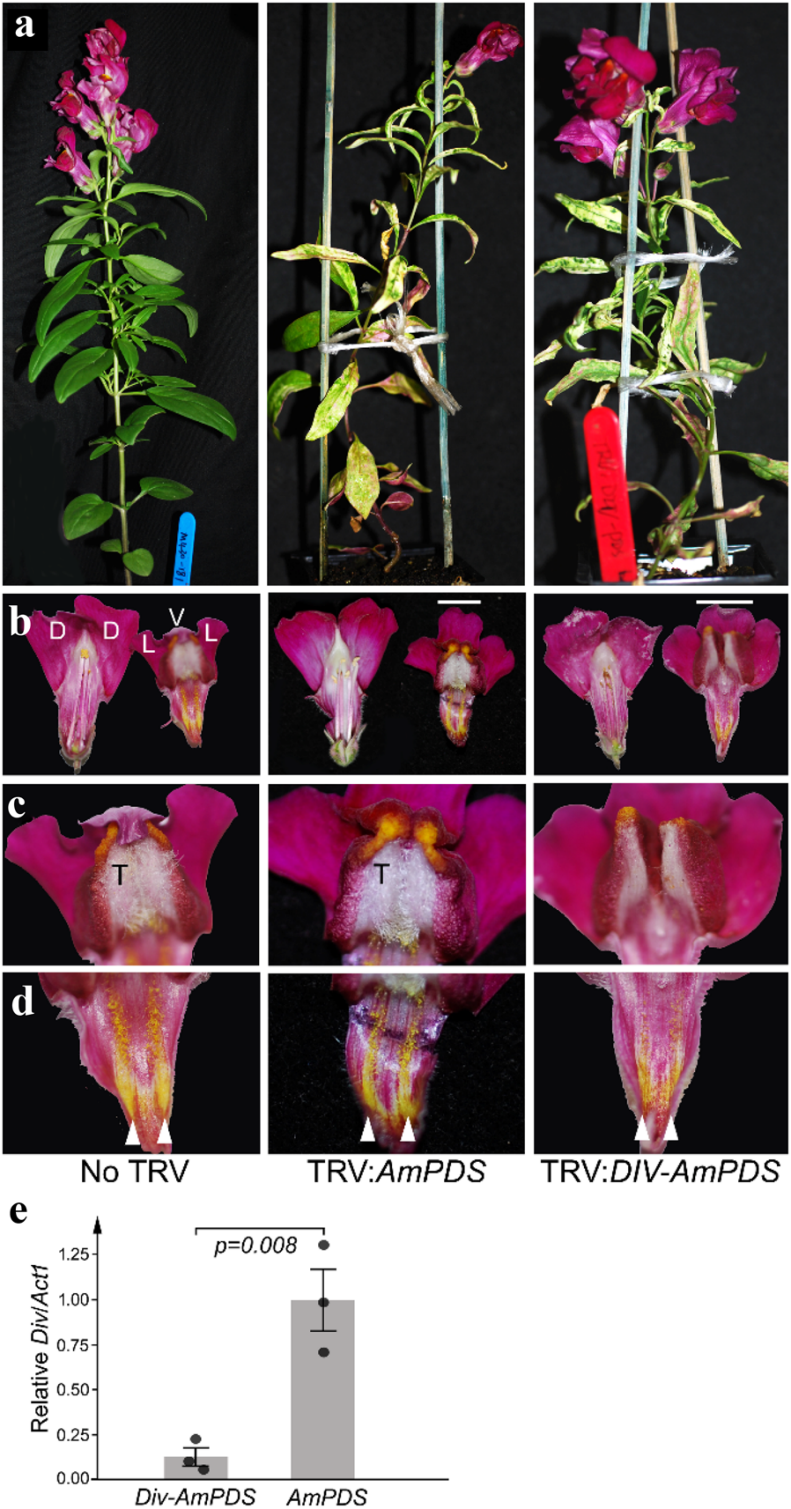
VIGS of the flower asymmetry gene *DIV*. **a** About one in five plants infected with virus carrying a fragment of *AmPDS* alone (middle column) or *DIV* and *AmPDS* (right column) showed bleaching of photosynthetic tissues throughout development. **b** The *Antirrhinum* corolla consists of two dorsal (D), two lateral (L) and one ventral (V) petal. In uninfected plants (left column) and plants with reduced *AmPDS* expression (middle), the ventral-most petal (shown with a white bar above it) folds to form the corolla face. **c** The ventral corolla face bears dense grey-coloured trichomes (T) internally. **d** The inside of the ventral corolla also has two stripes of yellow trichomes (nectar guides, arrowheads) that extend the full length of the corolla tube in uninfected or TRV:*AmPDS* infected plants. In TRV:*Div-AmPDS* infected plants (right column), folding or the ventral-most petal was reduced (b), internal grey trichomes were lost (**c**) and nectar guides were reduced (**d**). **e** Relative *Div* RNA abundance in young flower buds of plants infected with TRV:*Div-AmPDS* or TRV:*AmPDS*. Bars show the mean of three biological replicates (each a separate plant) ± their standard errors; the *p* value is from a Student’s t-test

To test the ability of VIGS to reduce expression of a flower-specific gene in *A. majus*, we targeted *Div*, which is expressed only during the early development of flowers where it is required for ventral identity of petals [38, 39]. A *Div* sequence of 275 bp was amplified from cDNA, its 3′ end joined to the 5′ end of the *AmPDS* sequence, and the fusion inserted into pTRV2sgP so that both sequences were in antisense orientation. The rationale for including *AmPDS* sequence was that it would allow VIGS to be monitored visually as bleaching. About one quarter of plants infected with virus carrying either *Div*-*AmPDS* or *AmPDS* without *Div* showed bleaching in the inflorescence stem, bracts and sepals (Fig. 3a). Of the 15 plants infected with the virus carrying the *Div*-*AmPDS* fusion, three had defects in ventral petals that included absence of the folded corolla face and of the dense pale hairs normally found inside it (Fig. 3b,c). The two strips of yellow hairs (nectar guides) inside the ventral corolla tube were also reduced in size (Fig. 3d; [38, 49]). These defects, which were present in multiple flowers of each plant, are characteristic of reduced Div activity [40] and were not seen in uninfected plants or those in which only *AmPDS* expression was reduced (first two columns in Fig. 3). Consistent with reduced Div activity, *Div* RNA abundance was lower in inflorescences with floral defects that had been infected with *Div-AmPDS* TRV, relative to those that had been infected with virus carrying *AmPDS* alone (Fig. 3e). These findings suggest that the VIGS method can be used to test the function of genes involved in *Antirrhinum* reproductive development.

**Fig. 4:**
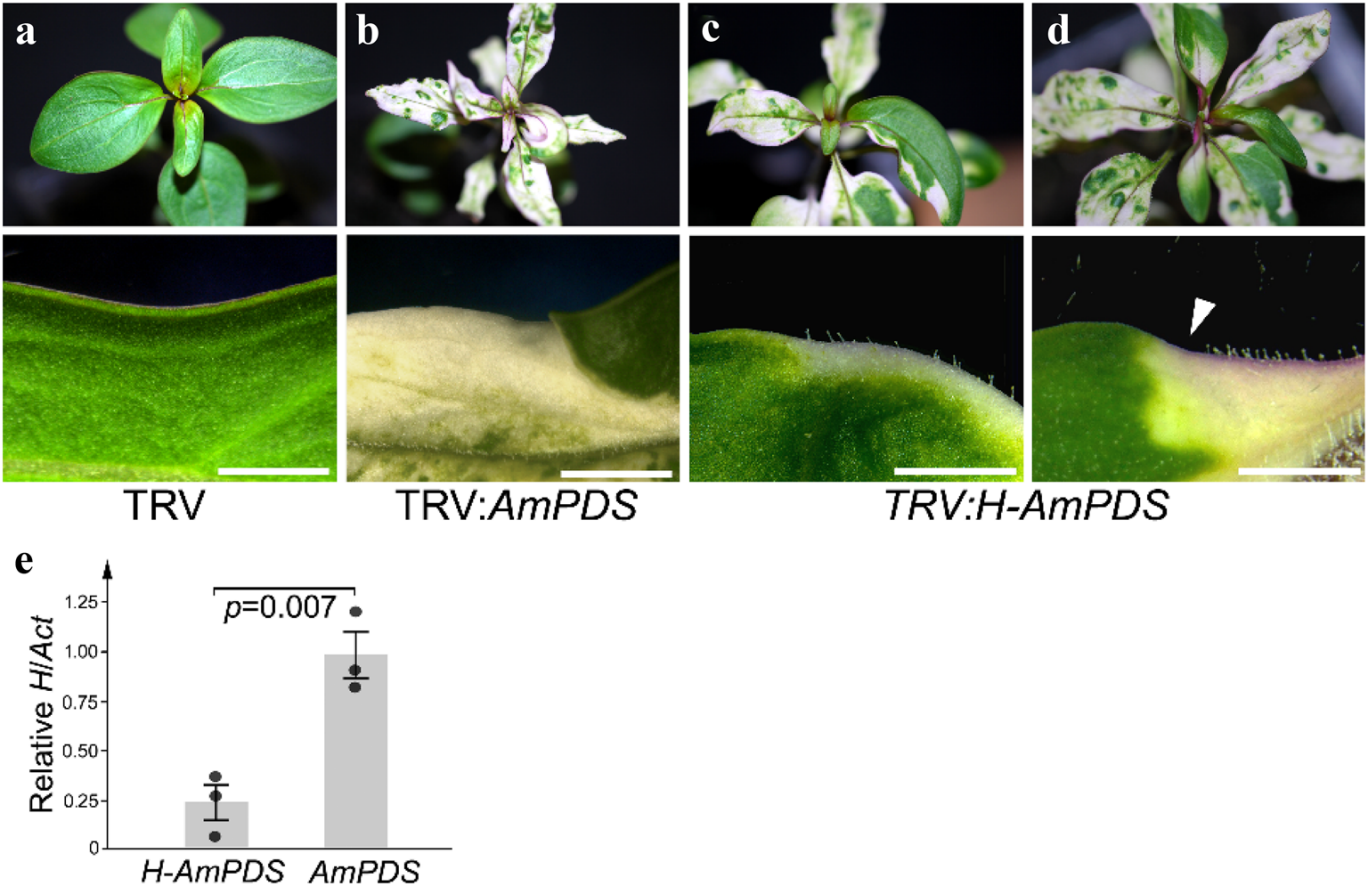
VIGS of the *H* gene. **a** Leaves above metamer 4 produce no epidermal hairs from the leaf blade in wild-type plants infected with empty TRV. **b** VIGS of *AmPDS* does not cause production of ectopic hairs. **c** ViGS of H causes ectopic trichomes to form in regions of the leaf blade also showing VIGS of *AmPDS*. **d** Not all bleached areas of leaf produce ectopic trichomes (arrowhead). Scale-bars show 5 mm. **e** Relative *H* RNA abundance in vegetative apices of plants infected with either TRV:*H-AmPDS* or TRV:*AmPDS*. Each bar shows the mean of three biological replicates (each a separate plant or pool of plants) ± its standard error; the *p*-value is from a Student’s *t*-test

**Fig. 5:**
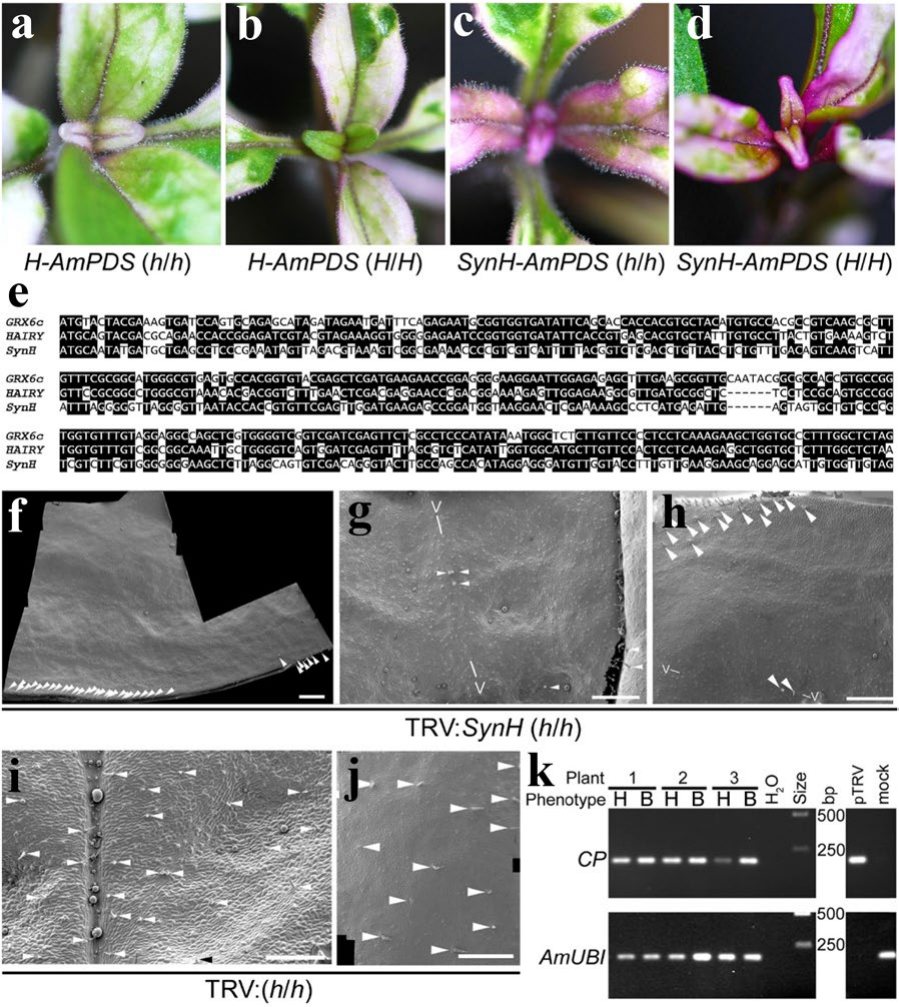
Complementation of the h mutant phenotype. **a** TRV carrying the wild-type *H* ORF and an *AmPDS* fragment failed to complement the h mutant phenotype (genotype *h*/*h*) and leaves remained hairy. **b** The same virus triggered VIGS of *H* and *AmPDS* in the wild-type plants (genotype *H*/*H*). **c** TRV with a synonymous H-encoding sequence (*SynH*) and *AmPDS* fragment also failed to complement h. **d** Unlike TRV with the wild-type *H* ORF, the *SynH* TRV did not cause VIGS of the wild-type *H* gene, though it triggered VIGS of *AmPDS*. The plants in **c** and **d** were grown together at a different time to those in (**a** and **c**). Differences in growth conditions, rather than the TRV, might therefore account for differences in purple anthocyanin expression. **e** Alignment of *SynH* with wild-type *H* and the most *H*-like gene (*GRX6c*) expressed in the *h*/*h* NIL. Identical nucleotides are boxed black. **f**–**h** TRV carrying the *SynH* sequence without *AmPDS*, led to leaf blades with large areas lacking trichomes in the *h* mutant NIL (remaining trichomes in the leaf blade are indicated by white arrowheads). Any trichomes that remained within the otherwise bald areas formed over major secondary veins (V). **i**, **j** Bald areas were not seen in the *h* mutant NIL infected with empty TRV. Scale bars in (**f**–**j**) show 1 mm. **k** TRV2 RNA was detected, using primers for its *CP* gene, in all bald (B) areas in *h* mutants and in corresponding hairy (H) areas from the same leaf or plant. Amplificiation of the *Ubiquitin1* (*AmUBI*) gene was used as a control for cDNA and pTRV2 and mock-inoculated plants as positive and negative controls for TRV detection, respectively

In contrast to *Div*, the *H* gene acts only in vegetative development. It is required to suppress trichome (hair) fate in the epidermis of leaf blades and stems above metamer 4, so loss of H activity allows formation of ectopic leaf blade and stem trichomes above this point, whereas the corresponding leaf blades and stems of wild-type are bald (Fig. 4a). Consistent with this role, *H* expression in leaves is confined to the developing epidermis (protoderm) of young primordia [41]. To attempt to silence *H*, 237 bp of its 3′-UTR was fused with the *AmPDS* fragment and inserted into pTRV2sgP so that the *H* fragment was in antisense orientation and *AmPDS* fragment in sense orientation. VIGS of *AmPDS* was seen in 97% of plants inoculated with virus carrying both sequences. In 58% of inoculated plants, ectopic hairs were seen within patches of bleached tissue in leaves above the metamer 4 (Fig. 4c, d), consistent with reduced H activity. Ectopic hairs were not seen in plants infected with TRV carrying *AmPDS* alone (Fig. 4b). The abundance of *H* RNA was reduced significantly in plants with ectopic hairs, even though RNA had been extracted from apices in which hair phenotypes were not yet visible (Fig. 4e). In contrast, previous work had shown that RNA from the most *H*-like transcript, *GRX6c* (Fig. 5a), was not affected by virus carrying the same *H* sequence used here to reduce H activity [41]. These results suggest that VIGS with TRV is also able to reduce the expression of genes that act early in leaf development and in the epidermis of *A. majus*.

### Reduced *AmPDS* expression as a marker for VIGS of *H*

While *AmPDS* prevents bleaching in photosynthetic tissues, *H* acts in the epidermis, in which only stomatal guard cells are photosynthetic. This raised the possibility that *H* might experience VIGS in leaves or stems that did not show VIGS of *AmPDS* internally (or vice versa) making bleaching an unreliable marker for VIGS of the second target gene. This proved not to be the case; ectopic hairs were noticed only when the underlying cells were bleached (Fig. 4c, d), suggesting that VIGS of *H* occurred in leaves where *AmPDS* expression was also reduced. However, not all bleached leaf tissue was associated with ectopic hairs (Fig. 4d). This might reflect differences in the level of VIGS experienced by *H* in the epidermis and by *AmPDS* in underlying cells, or a difference in the developmental stage at which the two genes are required (*H* in young primordia, *AmPDS* throughout leaf development). Nevertheless, reduced *PDS* activity appeared useful for narrowing down the tissues in which VIGS of the second target gene was likely to have occurred.

### Complementation of the *h* mutant phenotype

Several *Antirrhinum* species have lost H activity and produce hairs throughout development [41]. One of these species, *A. charidemi*, carries a null *h* mutation that produces no detectable transcript [41]. Its mutant *h* allele had been back-crossed into *A. majus* to replace a functional *H* allele and create a near-isogenic line (NIL) that produced hairs from all leaf blades and internodes. Because TRV was able to cause VIGS of *H* in the epidermis of young leaf primordia, we reasoned that it might also be capable of expressing H protein in these cells to complement the *h *mutant phenotype (*i.e.*, to suppress hair formation). We first tested this idea with the wild-type *H* open-reading frame (ORF). It was placed in sense orientation 3′ to the sgP of pTRV2sgP, which promotes production of sub-genomic RNA [32, 41]. We placed the *AmPDS* sequence in antisense orientation after the *H* stop codon, so that bleaching could be used to monitor virus infection. As expected, extensive VIGS of *AmPDS* was seen after inoculation of the *h* mutant NIL (Fig. 5a). However, we found no leaves or stems with reduced hair density to suggest that complementation of the *h* mutant phenotype had occurred. There were a number of explanations for the lack of complementation. One was that homology between the *H* sequence and a host transcript caused RNAi-mediated suppression of TRV, preventing sufficient H protein expression. Although the *H* ORF could cause VIGS of *H* in wild-type *A. majus* (Fig. 5b), a previous study had been unable to detect *H* RNA in the *h* mutant NIL [41], making homology between *H* transcripts an unlikely explanation. An alternative was that silencing had been triggered by homology between viral *H* RNA and the most *H*-like gene (*GRX6c*) that is expressed in the mutant NIL (73% nucleotide sequence identify, Fig. 5e). A third possibility was that the virus has been silenced via its *AmPDS* sequence, which caused extensive VIGS of the host *AmPDS* (Fig. 5a-d). A final possibility is that we had not included a consensus translation initiation (Kozak) sequence upstream of the H translation initiation codon.

To examine these possibilities, we replaced the *H* sequence in TRV2 with a synthetic sequence (*SynH*), which encoded wild-type H protein but carried synonymous substitutions that reduced its homology to both *H* (59% identity) and to *GRX6c* (57% identity; Fig. 5e). The *SynH* ORF was fused in-frame to a sequence encoding an antigenic FLAG-tag at its 3′ end and preceded by the wild-type *H* Kozak sequence. Infecting wild-type *A. majus* with this virus caused VIGS of *AmPDS*, as expected (Fig. 5d), though no ectopic trichomes were seen, suggesting that *H* and *SynH* lack sufficient homology for VIGS. Extensive VIGS of *AmPDS* was also seen in the *h* mutant NIL, indicating successful infection (Fig. 5c). However, we saw no reduction in hair density consistent with complementation of the *h* mutant phenotype. This suggested that homology between viral and host *AmPDS* sequences was responsible for suppression of viral RNA and consequently a lower level of TRV and recombinant protein expression. We therefore made a new TRV2 carrying the same *SynH-FLAG* ORF without *AmPDS*. When inoculated with this virus, about half of all *h* mutant plants (13 out of 30) produced leaves with areas largely lacking trichomes (Fig. 5f-h), as expected for complementation of the *h* mutant phenotype with virally-encoded H protein. Where trichomes were produced in these bald areas, they were rare and confined to the epidermis over secondary veins (*V* in Fig. 5g,h). Consistent with complementation, we detected TRV2 RNA in all bald areas of these plants (Fig. 5k). In contrast, we saw no similarly bald areas in an equivalent number of *h* mutant NILs infected with empty TRV2 (Fig. 5i-j). This strongly suggested that expression of H from TRV was sufficient to complement the *h* mutant phenotype. It also supported the idea that inclusion of *AmPDS* sequences had been responsible for lack of complementation by previous viral constructs. However, we could not exclude the possibility that the previous use of a wild-type *H* sequence had also contributed to the lack of complementation.

## Discussion

The VIGS method presented here proved a rapid way of reducing genes expression in *A. majus* and *M. orontium*, taking less than 1 month from initiation of experiments to plant infection and then from six days to 3 months for phenotypic effects to appear in vegetative shoots or flowers, respectively. This compares with a reported time of 5–7 months to regenerate the first generation of transgenic *A. majus* plantlets following infection with *A. tumefaciens* [10, 11]. The VIGS method was efficient, in that it led to reduced *AmPDS* activity in almost all inoculated plants and in the inflorescences of at least one in five plants inoculated as seedlings. It was also effective in all tissue layers—reducing activity of *H* in the epidermis and *AmPDS* internally–and in both the early stages of organ development (for *H* and *Div*) and later stages (for *AmPDS*).

Addition of an *AmPDS* sequence to a virus carrying a second target sequence allowed rapid visual identification of plants that were infected and revealed VIGS as tissue bleaching. It also identified plants in which VIGS persisted into reproductive development. We did not expect exact correspondence between tissues showing VIGS of *AmPDS* and production of ectopic trichomes as a result of reduced H activity, because the two genes are active in different tissue layers and at different stages of development. Despite this, bleaching did provide a useful indication of the regions that were likely to have experience VIGS of the second target gene (*H*) or, conversely, that were unlikely to have been affected and so could be used as an internal control in comparisons of phenotypes and gene expression. However, one disadvantage of using *AmPDS* as a marker for VIGS is that it reduced tissue and plant growth (compare, for example, the reduced growth in the half of the leaf showing bleaching in Fig. 4c, relative to the greener half of the same leaf). This makes it less suitable for testing the role of genes that affect growth, though this can be partly mitigated by use of control plants in which only *AmPDS* activity is reduced.

The TRV-VIGS method was also applied successfully to *M. orotinum*, providing a way to test evolutionary conservation of genes identified in *A. majus* [41]. Its use may extend to other members of the tribe *Antirrhineae*, allowing broader comparative studies.

TRV also proved a suitable vector for expressing the H protein to complement an *h* mutation. This was achieved using a synthetic *H* ORF containing numerous synonymous substitutions, although the extent to which reduced homology contributed to complementation is currently unclear. Compared to genetic transformation, in which a promoter can be chosen to give a particularly level or pattern of transgene expression, a disadvantage of TRV is that transgene expression is determined by viral infection and replication. Either of these factors could explain the inability of TRV carrying the H-encoding ORF to completely complement the *h* mutant phenotypes in cells over secondary veins, where *H* normally acts to suppress trichome fate in wild-type plants [41]. H is also a relatively small protein, as is GFP (107 and 239 amino acids, respectively), and we have not tested whether larger proteins can be expressed with the same efficiency.

Virus-mediated complementation has previously been reported only for tomato fruits [35, 36]. The method developed here opens up additional possibilities for functional genomics in *Antirrhinum* and its relatives. Potential uses include being able to test whether a gene is sufficient for a particular phenotype, including natural variation, and whether semi-dominance (which is common for natural variant alleles) reflects haplo-insufficiency. It also has the potential for use in biochemical and cell biological methods that are based on expression of proteins tagged with antigens or fluorescent proteins—for example, to identify protein locations within cells or to identify interacting proteins *in planta*.

## Conclusion

We describe an efficient system for VIGS in *Antirrhinum* and its relative *Misopates orontium*. In addition, we show that *PDS* silencing can be used as a visual marker for tissues experiencing reduced expression of a second target gene. We further demonstrate that TRV can be used as a vector for expression of a heterologous protein (GFP) in *Antirrhinum* and to complement a loss-of-function mutation. Together these techniques increase the scope for functional genomics in the model genus *Antirrhinum*.

## Methods

### Plant material and growth condition

All work involving virus-infected material was carried out in containment glasshouse, at 21.5 °C (± 1.0 °C) and a night-time temperature of 20± 0.2 °C. Supplemental lighting of 480 µmol m^−2^ s^−1^ intensity from metal halide lamps was used to maintain a 16 h day/8 h night cycle. To induce consistent germination, *A. majus* seeds were surface sterilized with ethanol and bleach according to the method of Manchado Rojo et al. [50] and sown onto 0.5 × Murashige and Skoog medium containing 3% sucrose [51]. Seedlings were transplanted individually into pots of John Innes No. 2 compost (Evergreen Garden Care Ltd, Frimley, UK) 10–14 days after sowing. All wild-type *A. majus* was the highly inbred line JI7. *Nicotiana benthamina* and *Misopates orontinum* seeds were sown directly onto compost and transplanted to pots of John Innes compost when large enough to handle.

### TRV constructs

The modified pTRV2 vector, lacking *2b* and *2c* genes and carrying a PEBV CP sgP was used here (Fig. 1a) [27]. The pTRV2sgP plasmid carrying a *GFP* ORF between its *Asc* I and *Bam* HI sites has been described previously [32]. A single *AmPDS* gene was identified with a tBlastn search of the *A. majus* reference genome [3] and a fragment comprising the last 200 bp of coding sequence and 163 bp of 3′-UTR was amplified from *A. majus* (JI.7) vegetative cDNA with primers AscI-PDS-F and AscI-PDS-R, containing *Asc* I sites (Table 2). The product was cloned into pJet 1.2 (ThermoFisher Scientific), excised and ligated in both orientations, into the *Asc* I site of pTRV2. To test the efficiency of VIGS at the flowering stage, a 275 bp fragment from the first exon of the *Div* gene was amplified from inflorescence cDNA of line JI.7 using primers AscI-DIV-R and PDS-DIVfusion-F (Table 2). This region was chosen because it was the least similar to parologous genes. The *Div* fragment fused to *AmPDS* by overlap PCR (see below) and inserted at the *Asc* I site of pTRV2sgP with both *Div* and *AmPDS* sequences in antisense orientation. For VIGS of *H*, 237 bp of its 3′-UTR were amplified from cDNA with primers AscI-Hutr-F and PDS-Hairyfusion-R (Table 2), fused tail-to-tail with the *AmPDS* fragment by overlap PCR and the fusion inserted into pTRV2sgP so that the *H* sequences was in antisense orientation and the *AmPDS* sequence in sense orientation.

**Table 2:** Oligonucleotide primers used in this study

Purpose	Primer name	Sequence (5′-3′)	Notes
VIGS	AscI-PDS-F	TGGCGCGCCCAATGAGCCTTACCGTGCAT	Adds *Asc* I sites to *AmPDS* fragment
	AscI-PDS-R	TGGCGCGCCGTAGTGCTTCAGAGGATCTTACAA	
	PDS-R	GTAGTGCTTCAGAGGATCTTAC	First-round *AmPDS* amplification
	AscI-DIV-R	AGGCGCGCCTACCCCATTCTAAGGTAAAAGG	Adds *Asc* I site to *Div* fragment
	PDS-DIVfusion-F	gtaagatcctctgaagcactacCATATTTTTCCAGCTCAAGCTGG	Adds PDS-R to *Div* fragment
	AscI-Hutr-F	AGGCGCGCCATAATACAAGTTGAGCAACAGCG	Adds *Asc* I site to *H* 3′-UTR fragment
	PDS-Hutrfusion-R	gtaagatcctctgaagcactacACAGAGTGATACGCCTCGAT	Adds PDS-R to *H* 3′-UTR fragment
H expression	AscI-Hairy-F	AGGCGCGCCATGCAGTACGACGCAGAACC	Adds *Asc* I site 5′ to the *H* ORF
	PDS-Hairyfusion-R	gtaagatcctctgaagcactacTTAGAGCCAAAGAGCACCAGC	Adds PDS-R 3′ to the *Hairy* ORF
	AscI-SynH-F	AGGCGCGCCAACAATGCAATATGATGCTGAGCCT	Adds *Asc* I upstream of *SynH* ORF
	FLAG-SynHfusion-R	CTA*CTTGTCGTCATCGTCTTTGTAGTC*CAACCACAATGCTCCTGCT	Replaces *H* stop with FLAG-tag
	FLAG-PDSfusion-R	*GACTACAAAGACGATGACGACAAG*TAGgtagtgcttcagaggatcttac	Adds FLAG to *AmPDS* fragment
RT-PCR	AmPDS-qPCR-F	TCTTTGTAATGGACGGCAAG	RT-PCR for *AmPDS*
	AmPDS-qPCR-R	ACTTGCCAAACTCTTCCCTG	
	TRV-CP-F	TGGGTTACTAGCGGCACTGA	RT-PCR for TRV2 RNA
	TRV-CP-R	GCTCGTCTCTTGAACGCTGA	
	AmUbi-qPCR-F	CCGAACCATCAGACAAACAAAC	RT-PCR for *AmUBI* control
	AmUbi-qPCR-R	TACCCTGGCCGACTACAATA	
	AmACT-F	CTTGGCCGTCTCCATTTCTT	RT-PCR for *AmACT* control
	AmACT-R	TCCTCACAGAGCGTGGATATAG	
	H-qPCR-F2	ACCATCCACAACACTCTAATC	RT-PCR for *H*
	H-qPCR-R	TGAATATCACCACCGGATTCTC	
	Div-F	GGGTAGTGGTCATGGATTCG	RT-PCR for *Div*
	Div-R	GGAAAGGAAGTTTGTGAATGGAG	

The *Asc* I restriction site, introduced to allow cloning into pTRV2sgP, is underlined. For overlap amplification of *AmPDS* fused to *H* or *AmDIV* sequences, *AmPDS* was first amplified with AscI-PDS-F and PDS-R and the *H* or *Div* sequence with one primer carrying an *Asc* I site and a reverse primer containing the complement of the PDS-R sequence at its 5′ end (lower case). Products were then fused by overlap PCR. To express SynH with a C-terminal FLAG-tag, the FLAG-encoding sequence (italics) was added in place of the SynH stop coding by amplification with FLAG-SynHfusion-R. To fuse the SynH-FLAG sequence to the *AmPDS* reporter, the *AmPDS* fragment was amplified with FLAG-PDSfusion-R, carring the FLAG-encoding sequence at its 5′ end

To fuse the *AmPDS* fragment to part of a second gene (Gene 2) by overlap PCR, an *Asc* I site was added to the 5′ end of the Gene 2 primer, and the sequence of the *AmPDS* primer, PDS-R, was added to the 5′ end of a second Gene 2-specific primer. These primers were used to amplify the Gene 2 fragment with an *Asc* I site at one end and *AmPDS* sequence at the other. The *AmPDS* sequence was amplified separately. All sequences were amplified from JI.7 cDNA using Q5 polymerase (New England Biolabs). The two PCR products were purified to remove primers using a NucleoSpin PCR clean-up kit (Macherey–Nagel) and then fused in a second round of PCR with primer AscI-PDS-F and the Gene 2-specific primers with an *Asc* I site. Fusion fragments were cloned into the *Eco* RV site of pJet 1.2, excised with *Asc* I and cloned into the *Asc I* site of pTRV2. The identity and orientation of inserts in recombinant plasmids was confirmed by Sanger sequencing.

To attempt expression of H protein from the wild-type *H* ORF, the ORF was amplified from JI.7 cDNA using primer AscI-Hairy-F, which introduced an *Asc* I site immediately upstream of the first ATG, and primer PDS-Hairyfusion-R after the stop codon for fusion to the *AmPDS* fragment by overlap PCR (Table 2). The fusion cloned into the *Asc* I site of pTRV2sgP with *H* in sense orientation and *AmPDS* in antisense orientation. Alternatively, a synthetic H-coding sequence (*SynH*) was used. This carried an *Asc* I site and the wild-type Kozak sequence 5′ to the initiation codon and had the H stop codon replaced with a sequence encoding the antigenic FLAG-tag, followed by a new stop codon. This was amplified with primers AscI-SynH-F and FLAG-SynHfusion-R and cloned into pJet 1.2, with the vector’s *Bgl* II site dowstream to the SynH-FLAG ORF. It was excised with *Asc* I and *Bgl* II and used to replace the *GFP* sequence between the *Asc* I and *Bam* HI sites of pTRV2sgP:GFP [32]. To express the *SynH-FLAG* ORF from a virus carrying the *AmPDS* reporter, the *AmPDS* fragment was amplified with primers FLAG-PDSfusion-R and AscI-PDSF, fused to the SynH-FLAG ORF, after its stop codon, by overlap PCR and the product inserted into the *Asc* I site of pTRVsgp with *SynH-FLAG* in sense orientation and *AmPDS* in antisense.

### VIGS assay in *Antirrhinum* and *Misopates*

The pTRV1 plasmid and recombinant pTRV2 plasmids were transferred to *A. tumefaciens* GV3101 pMP90 by freeze–thaw transformation [52] or electroporation [53]. Transformed *A. tumefaciens* was selected by culturing for two days at 28 °C on LB agar containing 50 µg/ml kanamycin sulfate, 25 µg/ml gentamycin sulfate and 50 µg/ml sodium rifampicin. Liquid starter cultures were grown overnight with shaking (120–180 rpm) in LB broth with the same concentrations of antibiotics, and 200 µl of the overnight culture used to inoculate 100 ml of LB with antibiotics. After a further ~ 24 h, cells were pelleted and resuspended in freshly prepared infiltration medium (10 mM MES pH 5.6, 10 mM MgCl_2_, 150 µM acetosyringone) to a final OD_600_ of 1.5 and incubated at room temperature for 3 h [53]. A 1:1 mixture of cells carrying pTRV1 or pTRV2 was used to infiltrate leaves of 3 week-old *N. benthamiana* plants by the needle-less syringe method [17]. After 5–7 days, leaves higher on the plant showing symptoms of infection (distorted shape and less chlorophyll) were ground in phosphate buffer (0.577 mM Na_2_HPO_4_, 0.423 mM NaH_2_PO_4,_ pH 7.0) on ice [54]. To avoid any effects of variation between batches of viral sap when comparing the effects of inoculation at different developmental stages, each comparison was made using the same batch of *N. benthamiana* sap, either freshly prepared or thawed on ice after storage at -80 °C.

*A. majus* and *M. orontium* were inoculated by scattering insoluble Al_2_O_3_ grit (Sigma, particle size > 0.63 mm) over leaves or cotyledons, pipetting sap onto them (10 µl onto each organ) and rubbing between gloved finger and thumb until chlorophyll was seen to bleed into the buffer [55]. Although Al_2_O_3_ and sap were added to the adaxial surface of leaves and cotyledons, they became distributed on both sides of the organ during rubbing. Plants were then covered with transparent plastic lids for 3 days, to maintain a high relative humidity.

### TRV-mediated protein expression in *Antirrhinum*

The *h* mutant NIL used to test complementation by TRV-expressed H protein had been generated previously by introgressing the *h* mutant allele from *A. charidemi* into the genetic background of *A. majus*, by repeated back-crossing [41]. Plants were infected with either empty or recombinant TRV2, together with TRV1, and examined 6–10 weeks after inoculation for effects on trichome phenotypes. Trichome phenotypes were documented by scanning electron microscopy, as described previously [41].

### RNA extraction and qRT-PCR

To quantify *AmPDS* or *H* RNA, three shoot apices in which the youngest visible leaves were at metamer 4–5 were pooled for each of three biological replicates, while for *Div* expression young flower buds (< 3 mm) were used. Tissues were immediately frozen in liquid nitrogen on harvest. Total RNA was extracted using TRIzol reagent (Invitrogen) and purified directly on a Purelink silica column (ThermoFisher Scientific) [33]. RNA samples were treated with RQI DNase (Promega Corporation) to remove genomic DNA and 0.5 µg of RNA then reverse transcribed to cDNA using M-MLV Reverse Transcriptase (Promega Corporation) according to the supplier’s instructions, with random hexamers used as primers. Quantitative RT-PCR was performed using LightCycler® 480 SYBR Green I master mix (Roche Life Science) in a LightCycler 96 instrument (Roche Life Science). Either the *Ubiquitin1* gene (*AmUBI*) or *AmACTIN* (*ACT*) were used for relative quantification of expression [13]. Amplification efficiencies for each target gene were estimated by amplifying a tenfold dilution series of pooled cDNA templates, and the efficiency value used to calculate the relative abundance of the target cDNA in each sample from its Cp value. To avoid amplifying cDNAs originating from TRV2 RNA, at least one of the primers in each PCR was complementary to a sequence outside the region present in pTRV2. Primers used for qRT-PCR are listed in Table 2. To detect viral RNA in complemented *h* mutants, RNA was extracted from largely bald areas of leaves and from corresponding hairy areas of the same leaf or an opposite leaf. In these cases, cDNA was amplified with primers for the TRV2 *CP* gene or *AmUBI* (Table 2). For *Div*, RNA was extracted from flower buds (< 3 mm) and expression was estimated by quantifying PCR products in agarose gels after staining with ethidium bromide (500 ng ml^−1^). In this case, a two-fold serial dilution of the cDNA sample with the most abundant template (from 1:2 to 1:128) was amplified alongside test samples. Fluorescence was quantified in Fiji [56] from fluorescence images captured within the camera’s dynamic range, and values from the serial dilution regressed onto log_2_ of relative template abundance to confirm a close relationship between product and template abundance (*R*^*2*^ ≥ 0.97) and that all PCRs were template-limited. Relative abundance of the template in test samples was interpolated from this regression.

For all genes, the mean of the ratios of the target cDNA to *ACT* or *UBI* in each treatment was calculated for at least three biological replicates, each a different plant or group of plants. Differences in means of relative abundance were detected with *t*-tests or ANOVA and Tukey post-hoc tests.

## Data Availability

All data generated or analysed during this study are included in this published article.
